# Author Correction: A circRNA signature predicts postoperative recurrence in stage II/III colon cancer

**DOI:** 10.1038/s44321-026-00510-9

**Published:** 2026-08-25

**Authors:** Huai-Qiang Ju, Qi Zhao, Feng Wang, Ping Lan, Zixian Wang, Zhi-Xiang Zuo, Qi-Nian Wu, Xin-Juan Fan, Hai-Yu Mo, Li Chen, Ting Li, Chao Ren, Xiang-Bo Wan, Gong Chen, Yu-Hong Li, Wei-Hua Jia, Rui-Hua Xu

**Affiliations:** 1 State Key Laboratory of Oncology in South China, Collaborative Innovation Center for Cancer Medicine, Sun Yat-sen University Cancer Center, Guangzhou, China; 2 Department of Medical Oncology, Sun Yat-sen University Cancer Center, Guangzhou, China; 3 The Sixth Affiliated Hospital, Sun Yat-sen University, Guangzhou, China; 4 State Key Laboratory of Biocontrol, School of Life Sciences, Sun Yat-sen University, Guangzhou, China; 5 Department of Pathology, Sun Yat-sen University Cancer Center, Guangzhou, China; 6 Department of Colorectal Surgery, Sun Yat-sen University Cancer Center, Guangzhou, China

## Abstract

**Figure:**
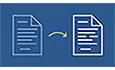

**Correction to:** *EMBO Molecular Medicine* 11:e10168. https://doi.org/10.15252/emmm.201810168 | Published online 2 September 2019

The authors contacted the journal after identifying errors in Figures 4C and 4G of the published manuscript. The authors supplied raw data confirming the error, and the journal agrees to correct Figures 4C and 4G.

**Figure:**
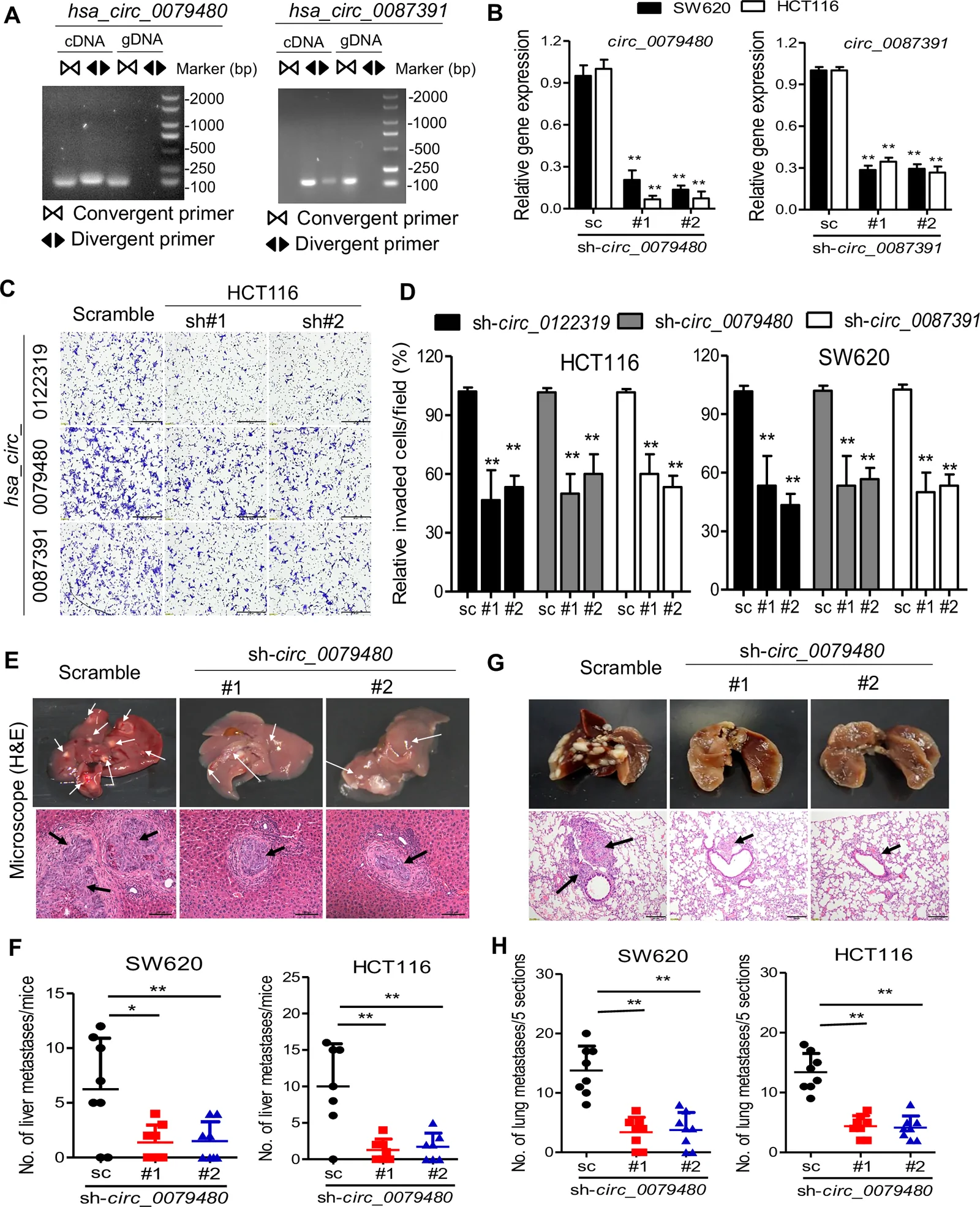
Figure 4. Original. Source data are available online for this figure

**Figure:**
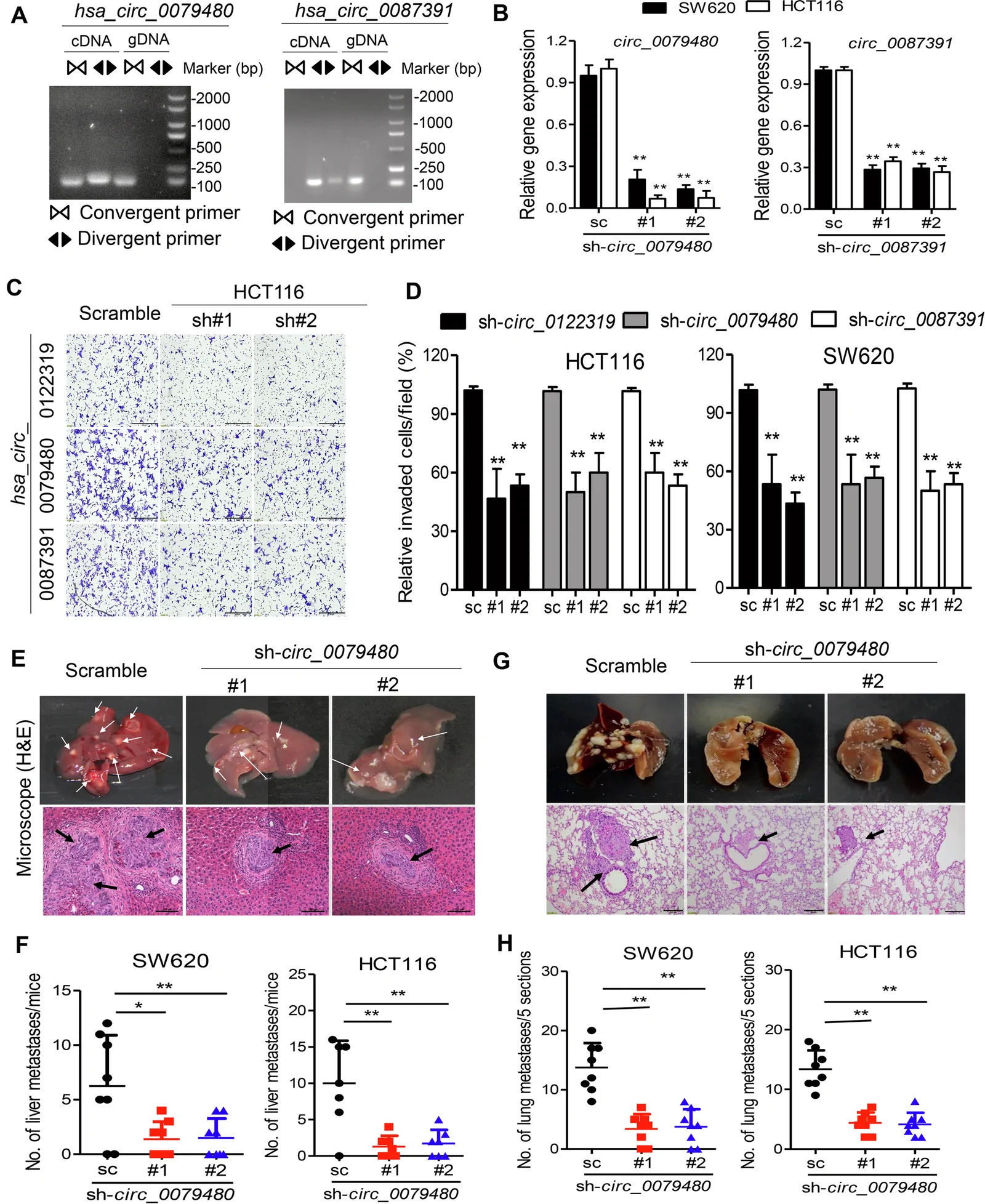
Figure 4. Corrected. Source data are available online for this figure

**Figures 4C and 4G are withdrawn and replaced**

Author statement:

The authors regret that incorrect images were used in Figure 4 during data arranging. The representative image of the migration assay for the hsa_circ_0079480-sh#2 group in Figure 4C, and the H&E-stained lung section for the hsa_circ_0079480-sh#2 group in Figure 4G, were incorrectly displayed.

The authors state that the results and conclusions of the article remain unchanged. The authors apologize for the oversight and any inconvenience it may have caused.

This correction does not affect the interpretation or conclusions of the study.

All authors agree to this correction.

## Supplementary information

Figure 4 source-data

