# Supplementary material for: A circRNA signature predicts postoperative recurrence in stage II/III colon cancer
Source: EMBO Mol Med. 2019 Sep 2;11(10):e10168. doi: 10.15252/emmm.201810168 (PMC6783650; doi:10.15252/emmm.201810168)
Supplement: Supplementary file 1 — Appendix [file EMMM-11-e10168-s001.pdf]

Appendix

A circRNA signature predicts postoperative recurrence in stage II/III colon cancer

Huai-Qiang Ju, Qi Zhao, Feng Wang, Ping Lan, Zixian Wang, Zhi-Xiang Zuo, Qi-Nian Wu, Xin-Juan Fan, Hai-Yu Mo, Li Chen, Ting Li, Chao Ren, Xiang-Bo Wan, Gong Chen, Yu-Hong Li, Wei-Hua Jia, Rui-Hua Xu

Appendix Table

Appendix Table S1. Characteristics of 4 circRNA markers and their coefficients in stage II/III colon cancer prognosis prediction of disease free survival.

Appendix Table S2. The final Cox regression model for prediction of disease-free survival.

Appendix Table S3. The final Cox regression model for prediction of overall survival.

Appendix Table S4. The circRNA ID, gene sympol, divergent primer and Back-splice junction (BSJ) coordinate for 22 circRNAs.

Appendix Table S5. *P* values for differences between experimental groups in figures.

Appendix Table S1:

Characteristics of 4 circRNA markers and their coefficients in stage II/III colon cancer prognosis prediction of disease free survival.

CircRNA	ID	Coefficient	HR (%CI)	*P* value
**hsa_circ_0122319**	0.4604	1.5847	(1.1020-2.2789)	0.013
**hsa_circ_0008039**	-0.3862	0.6796	(0.5602-0.8246)	<0.001
**hsa_circ_0079480**	0.2929	1.3402	(1.0140-1.7714)	0.04
**hsa_circ_0087391**	0.4386	1.5505	(1.1516-2.0877)	0.004

HR: hazard ratio. CI=confidence interval. CircRNA=Circular RNA

Appendix Table S2:

The final Cox regression model for prediction of disease-free survival.

	**HR (95% CI)**	***P* value**
CirScore (high vs. low)	4.67 (2.66–8.20)	<0.0001
Age (≥65 vs. <65)	1.49 (0.91–2.45)	0.1153
N stage (N1 vs. N0)	1.51 (0.84–2.73)	0.1686
N stage (N2 vs. N0)	2.79 (1.54–5.05)	0.0007
NI (yes vs. no)	2.91 (1.78–4.76)	<0.0001
VI (yes vs. no)	3.28 (1.99–5.39)	<0.0001

HR=hazard ratio. CI=confidence interval. NI=perineural invasion. VI=lymphatic or vascular invasion.

Appendix Table S3:

The final Cox regression model for prediction of overall survival.

	**HR (95% CI)**	***P* value**
CirScore (high vs. low)	5.45 (2.70–11.00)	<0.0001
Age (≥65 vs. <65)	1.61 (0.90–2.89)	0.1110
N stage (N1 vs. N0)	2.64 (1.30–5.37)	0.0074
N stage (N2 vs. N0)	3.68 (1.78–7.65)	0.0005
NI (yes vs. no)	2.64 (1.49–4.66)	0.0008
VI (yes vs. no)	2.30 (1.27–4.16)	0.0058

HR=hazard ratio. CI=confidence interval. NI=perineural invasion. VI=lymphatic or vascular invasion.

Appendix Table S4:

The circRNA ID, gene sympol, divergent primer and Back-splice junction (BSJ) coordinate for 22 circRNAs.

CircRNA ID*	Gene symbol	Forward primer	Reverse primer	BSJ (hg19)	Previous reports
hsa_circ_0122319	NM_182943;PLOD2	tgaaagaagtcatggaacac	aatcgatggaatccatcact	chr3:145838898:145842016	\
hsa_circ_0008039	NM_001164761;PRKAR1B	acgccgtgtcctacgtcag	tggctgcttccttcctcctc	chr7:716866:751164	(Liu et al, 2018)
hsa_circ_0079480	NM_001101426;ISPD	gcaaatcatcttagatcaatgc	tcttgggaaattctcttgaaac	chr7:16298015:16317851	\
hsa_circ_0087391	NM_001286715;AGTPBP1	tccagtcttataaaattatctgc	cattgatcttctccagttga	chr9:88284400:88327481	\
hsa_circ_0016600	NM_001373;DNAH14	ctcatacctactttggaatggc	ctataaaggaactggctgctc	chr1:225140372:225161855	\
hsa_circ_0016601	NM_001373;DNAH14	gtacttttctaaagccagttc	tctctaatggtttcacatcttc	chr1:225140372:225195246	\
hsa_circ_0003500	NM_001244898;PTBP3	ctcctattactcctcaccttcg	tcatcagatccccgagcttg	chr9:115030329:115060196	\

hsa_circ_0073762	NR_046207;SLC12A2	gaacaacttccaggaggagc	ccttaagcaactccaccacg	chr5:127466759:127477673	\
hsa_circ_0004887	NM_198433;AURKA	atgctaccagagtctacct	ctcaggattattttagtagcag	chr20:54956489:54959380	\
hsa_circ_0005598	NM_001009;RPS5	agatcatacacctgctcaca	ctgcaatgtaatcctggttc	chr19:58904343:58904854	\
hsa_circ_0001944	NR_026975;FIRRE	ggtctgttcccaatacaagaag	ctgacaccttagtctcctcata	chrX:130883334:130928494	(Fu et al, 2018)
hsa_circ_0078607	NM_021977;SLC22A3	gctgattactcggaagaaag	agacaaggtcaaactctgag	chr6:160819011:160831878	\
hsa_circ_0000778	NM_152347;EFCAB13	tggtatacctgatttggagc	tgtactttgtcaccttccttg	chr17:45479498:45492285	\
hsa_circ_0006618	NM_014933;SEC31A	gtgaacattttccagcaggatg	catccaattgctgagcagatg	chr4:83799883:83803093	\
hsa_circ_0001064	NM_020909;EPB41L5	gttgatatgcatgtggtcaag	gatgctttttgtaccatccaac	chr2:120830754:120836157	\
hsa_circ_0067582	NM_001303246;RASA2	agcttgttgtacagtgaagc	ctggaaagttcttggaatctc	chr3:141231005:141259451	\
hsa_circ_0004270	NM_013386;SLC25A24	ggcatatgaacagtacaagaag	catcaacatcaatgctccattg	chr1:108690901:108703915	\
hsa_circ_0008336	NM_198428;BBS9	gagctgtacaatttcgggccattc	aactcgcggaatgcctgcttg	chr7:33397467:33427756	\
hsa_circ_0004689	NM_017673;SWT1	gcagctgtgagggatattc	gaccttgtactgaaagcatg	chr1:185183639:185200840	\

hsa_circ_0001824	NM_001247996;ASAP1	ttataggtccttggtcaag	cagttatgactaataatttatcattc	chr8:131164982:131181313	\
hsa_circ_0025967	NM_004719;SCAF11	cgtgagaaagactttgccag	cttgcaagaagtaccctgtc	chr12:46319925:46322642	\
hsa_circ_0079534	NM_182762;MACC1	gatgttgctgttgaggtgga	gccaattgtgaagcaagtctg	chr7:20193816:20201493	\

REFERENCES

Fu B, Zhang A, Li M, Pan L, Tang W, An M, Liu W, Zhang J (2018) Circular RNA profile of breast cancer brain metastasis: identification of potential biomarkers and therapeutic targets. *Epigenomics* **10:** 1619-1630

Liu Y, Lu C, Zhou Y, Zhang Z, Sun L (2018) Circular RNA hsa_circ_0008039 promotes breast cancer cell proliferation and migration by regulating miR-432-5p/E2F3 axis. *Biochem Biophys Res Commun* **502:** 358-363

Appendix Table S5.

***P* values for differences between experimental groups in figures.**

Figure 2C

	**Student's t-test**		**Significant?**	**Summary**	***P value***
hsa_circ_0122319	T	N	Yes	**	<0.0001
hsa_circ_0087391	T	N	Yes	**	<0.0001
hsa_circ_0079480	T	N	Yes	**	<0.0001
hsa_circ_0008039	T	N	Yes	**	<0.0001

Figure 4B

	**Student's t-test**		**Significant?**	**Summary**	***P value***
hsa_circ_0079480	SW620-sc	#1	Yes	**	0.0051
hsa_circ_0079480	SW620-sc	#2	Yes	**	0.0035
hsa_circ_0079480	HCT116-sc	#1	Yes	**	0.0056
hsa_circ_0079480	HCT116-sc	#2	Yes	**	0.0078
hsa_circ_0087391	SW620-sc	#1	Yes	**	0.0048
hsa_circ_0087391	SW620-sc	#2	Yes	**	0.0052
hsa_circ_0087391	HCT116-sc	#1	Yes	**	0.0034
hsa_circ_0087391	HCT116-sc	#2	Yes	**	0.0047

Figure 4D					
	**Student's t-test**		**Significant?**	**Summary**	***P value***
hsa_circ_0122319	HCT116-sc	#1	Yes	**	0.0039
hsa_circ_0122319	HCT116-sc	#2	Yes	**	0.0081
hsa_circ_0079480	HCT116-sc	#1	Yes	**	0.0092
hsa_circ_0079480	HCT116-sc	#2	Yes	**	0.0021
hsa_circ_0087391	HCT116-sc	#1	Yes	**	0.0021
hsa_circ_0087391	HCT116-sc	#2	Yes	**	0.0002
hsa_circ_0122319	SW620-sc	#1	Yes	**	0.0057
hsa_circ_0122319	SW620-sc	#2	Yes	**	<0.0001
hsa_circ_0079480	SW620-sc	#1	Yes	**	0.0056
hsa_circ_0079480	SW620-sc	#2	Yes	**	0.0002
hsa_circ_0087391	SW620-sc	#1	Yes	**	0.0009
hsa_circ_0087391	SW620-sc	#2	Yes	**	0.0002

Figure 4F					
	**Student's t-test**		**Significant?**	**Summary**	***P value***
hsa_circ_0079480	SW620-sc	#1	Yes	*	0.0114
hsa_circ_0079480	*SW620-sc*	*#2*	Yes	**	0.0097
hsa_circ_0079480	HCT116-sc	#1	Yes	**	0.0025
hsa_circ_0079480	HCT116-sc	#2	Yes	**	0.0039

Figure 4H					
	Student's t-test		Significant?	Summary	***P value***
hsa_circ_0079480	SW620-sc	#1	Yes	**	<0.0001
hsa_circ_0079480	SW620-sc	#2	Yes	**	<0.0001
hsa_circ_0079480	HCT116-sc	#1	Yes	**	<0.0001
hsa_circ_0079480	HCT116-sc	#2	Yes	**	<0.0001

Figure EV1A					
	Student's t-test		Significant?	Summary	***P value***
hsa_circ_0016600	N	T	Yes	**	<0.0001
hsa_circ_0016601	N	T	Yes	**	<0.0001
hsa_circ_0003500	N	T	Yes	**	<0.0001
hsa_circ_0001944	N	T	Yes	**	<0.0001
hsa_circ_0078607	N	T	Yes	**	<0.0001
hsa_circ_0004887	N	T	Yes	*	0.0408
hsa_circ_0079534	N	T	Yes	**	<0.0001
hsa_circ_0005998	N	T	Yes	**	0.0005
hsa_circ_0073762	N	T	Yes	**	<0.0001

Figure EV1B					
	Student's t-test		Significant?	Summary	***P value***
hsa_circ_0000778	N	T	Yes	**	<0.0001

hsa_circ_0006618	N	T	Yes	**	<0.0001
hsa_circ_0008336	N	T	Yes	**	0.0011
hsa_circ_0004270	N	T	Yes	**	<0.0001
hsa_circ_0001046	N	T	Yes	**	<0.0001
hsa_circ_0004689	N	T	Yes	*	0.0052
hsa_circ_0067582	N	T	Yes	**	<0.0001
hsa_circ_0001842	N	T	Yes	**	<0.0001
hsa_circ_0025967	N	T	Yes	**	0.0022

Figure EV2B

	Student's t-test		Significant?	Summary	*P value*
hsa_circ_0122319	circRNA	mRNA	Yes	**	<0.0001
hsa_circ_0087391	circRNA	mRNA	Yes	**	<0.0001
hsa_circ_0079480	circRNA	mRNA	Yes	**	<0.0001
hsa_circ_0008039	circRNA	mRNA	Yes	**	<0.0001

Figure EV3C

	Student's t-test		Significant?	Summary	*P value*
hsa_circ_0122319	SW620-sc	#1	Yes	**	0.0039
hsa_circ_0122319	SW620-sc	#2	Yes	**	0.0015
hsa_circ_0122319	HCT116-sc	#1	Yes	**	0.0094
hsa_circ_0122319	HCT116-sc	#2	Yes	**	0.0095
