# Supplementary material for: A circRNA signature predicts postoperative recurrence in stage II/III colon cancer
Source: EMBO Mol Med. 2019 Sep 2;11(10):e10168. doi: 10.15252/emmm.201810168 (PMC6783650; doi:10.15252/emmm.201810168)
Supplement: Supplementary file 2 — Expanded View Figures PDF [file EMMM-11-e10168-s002.pdf]

Expanded View Figures

Figure EV1. Expression status of candidate circRNAs as colon cancer recurrence-specific biomarkers.

A qRT–PCR validation of the nine selected upregulated circRNAs in an independent validation cohort of 48 pairs of colon cancer tissues.
B qRT–PCR validation of nine selected upregulated circRNAs in an independent validation cohort of 48 colon cancer tissues with or without recurrence.

Data information: The horizontal bar represents mean expression levels; N = adjacent normal tissues; T = tumor tissues; R = tumor tissues from the recurrence group. $^{**}P < 0.01$, Student's *t*-test, mean ± SD. Exact *P*-values are specified in Appendix Table S5.

© 2019 The Authors

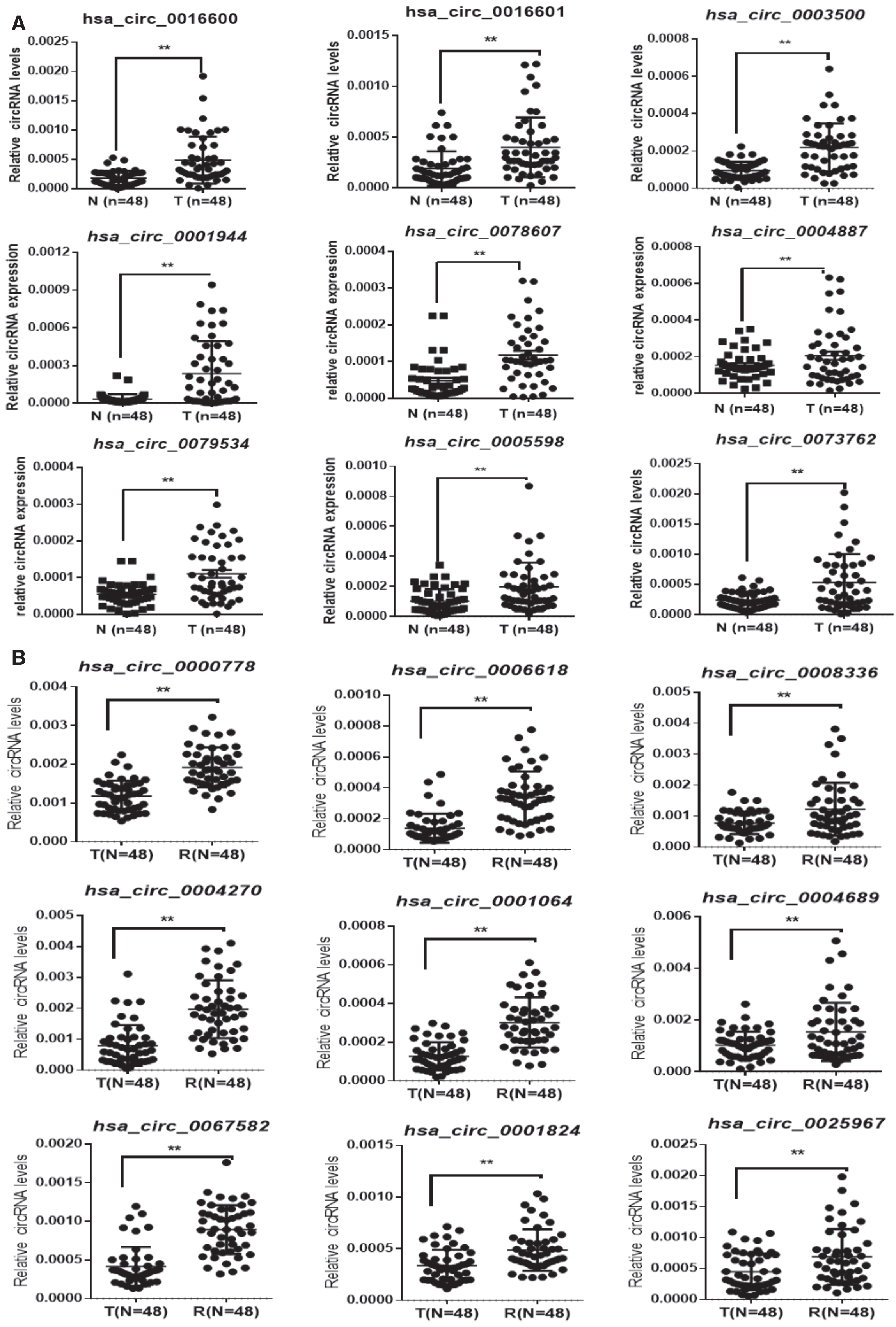
A
hsa_circ_0016600
hsa_circ_0016601
hsa_circ_0003500
hsa_circ_0001944
hsa_circ_0078607
hsa_circ_0004887
hsa_circ_0079534
hsa_circ_0005598
hsa_circ_0073762
Relative circRNA levels
Relative circRNA expression
relative circRNA expression
N (n=48)
T (n=48)
**
B
hsa_circ_0000778
hsa_circ_0006618
hsa_circ_0008336
hsa_circ_0004270
hsa_circ_0001064
hsa_circ_0004689
hsa_circ_0067582
hsa_circ_0001824
hsa_circ_0025967
Relative circRNA levels
T(N=48)
R(N=48)

Figure EV1.

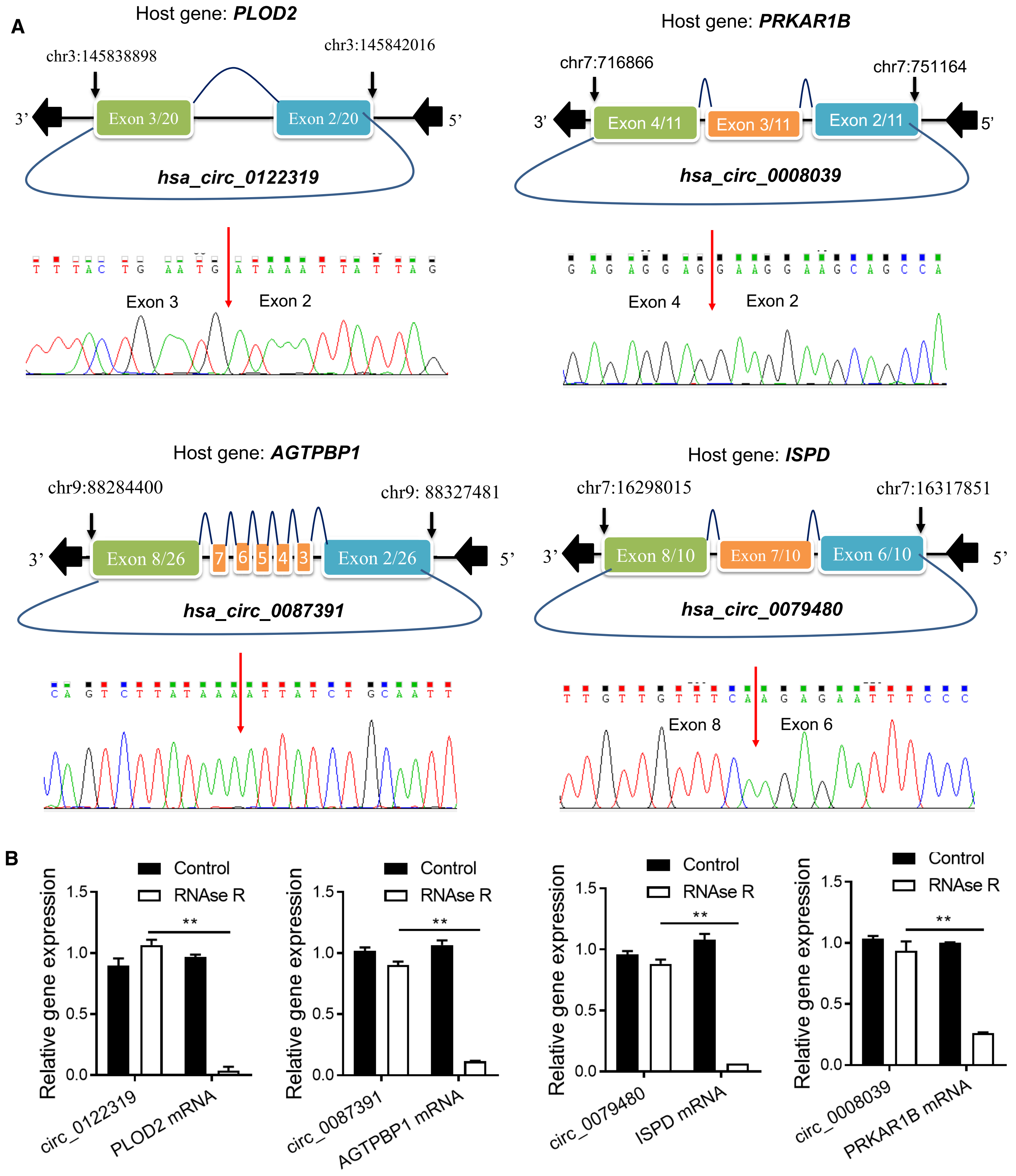

Figure EV2. Characterization of selected circRNAs.

A The genomic locus of the selected circRNAs, and the circRNA back-splice junction sites were detected by RT–PCR followed by Sanger sequencing.

B qRT–PCR analysis for the expression of four selected circRNAs and the corresponding host genes after treatment with RNase R in HCT116 cells. The data were normalized to mouse GAPDH mRNA by adding a mouse RNA spike to each fraction. ***P* < 0.01, Student's *t*-test, mean ± SD (*n* = 3). Exact *P*-values are specified in Appendix Table S5.

© 2019 The Authors

Figure EV3. Characterization of selected circRNAs.

A qRT–PCR analysis of four circRNA expression in 11 colon cancer cell lines and 2 colon epithelial cells (CCD112 and CCD841). Data are presented as mean ± SD (n = 3).

B RT–PCR products with divergent and convergent primers showing circularization of has_circ_0122319. cDNA, complementary DNA; gDNA, genomic DNA.

C qRT–PCR evaluated the knockdown efficiency of has_circ_0122319 and has_circ_0097391 and the host gene (PLOD2 and AGTPBP1) expression in SW620 and HCT116 cells transfected with two unique shRNAs (#1, #2). **$P < 0.01$, Student's t-test, mean ± SD (n = 3). Exact P-values are specified in Appendix Table S5.

D qRT–PCR evaluated the host gene (PLOD2, AGTPBP1, and ISPD) mRNA expression, and immunoblotting evaluated the PLOD2 protein expression in indicated cells. β-Actin antibody was used as a loading control. Data are presented as mean ± SD (n = 3).

E Correlation analysis of transcriptome between two independent shRNA groups in SW620 and HCT116 cells. In each scatter plot, the log10-transformed Transcripts Per Million (TPM) of reads of each gene were utilized for calculating the spearman's coefficient. R represents Spearman's correlation coefficients, and P-values were calculated by Spearman's correlation test.

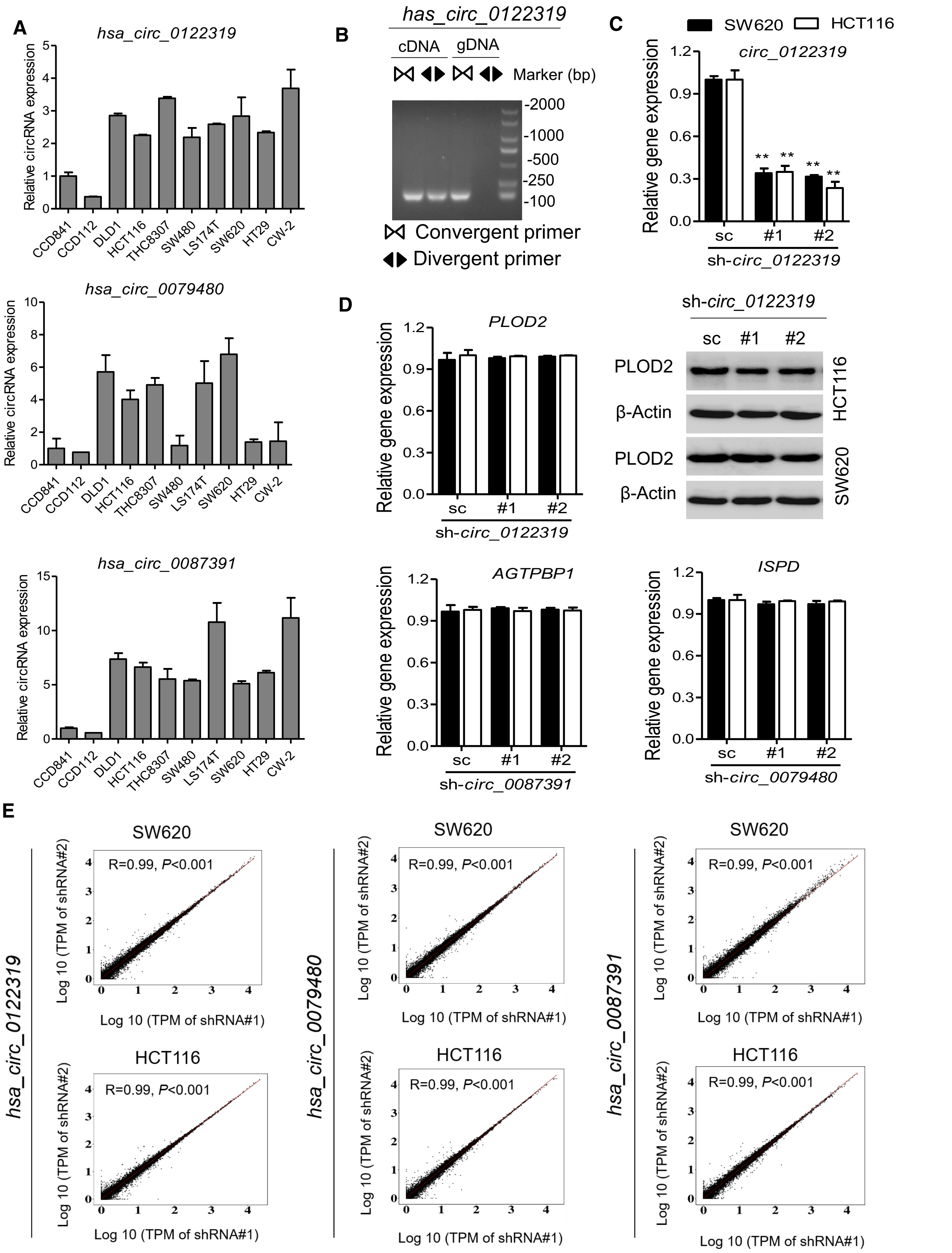

Figure EV3.

© 2019 The Authors

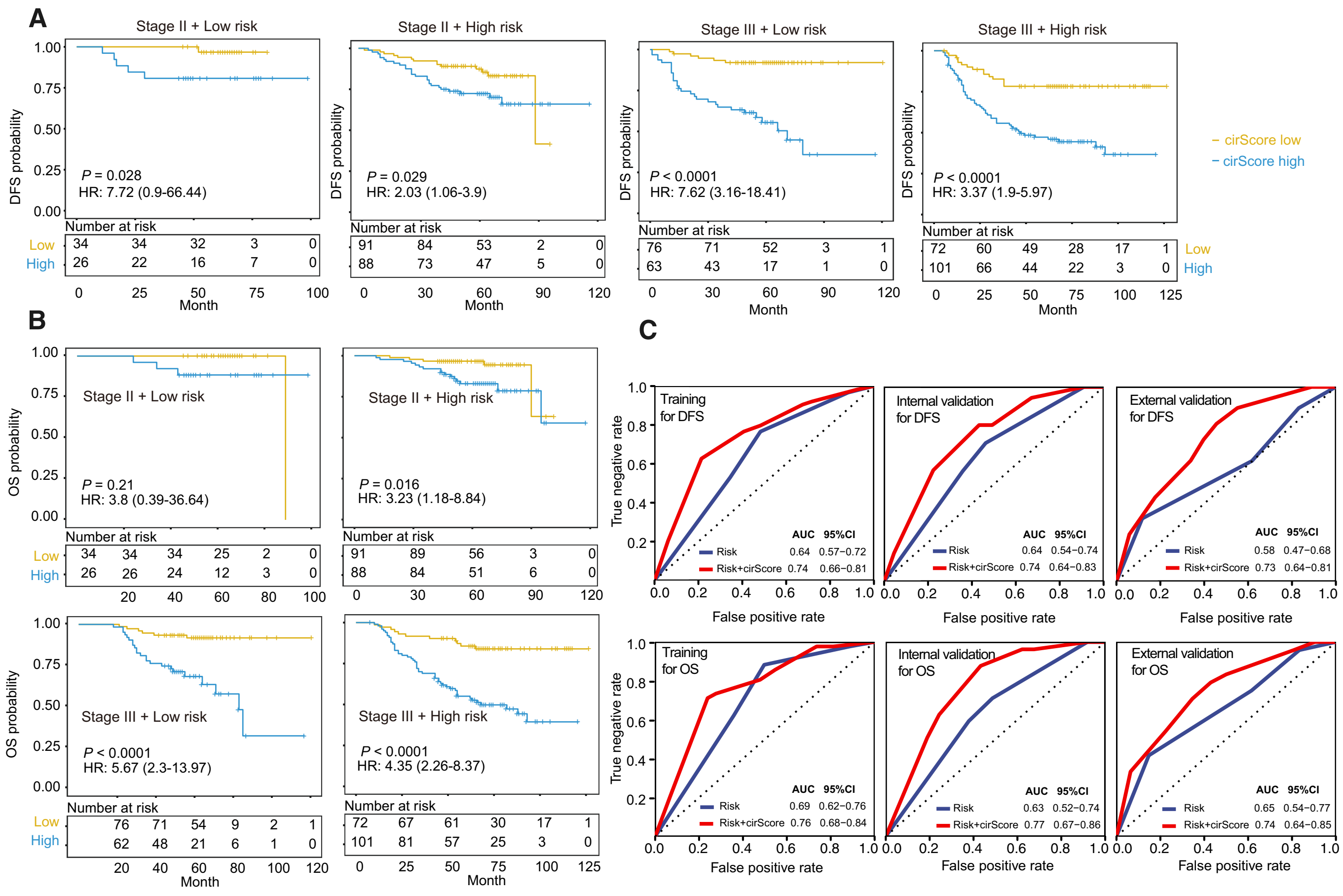

Figure EV4. Stratified analysis with known risk factors.

A The impact of the cirScore on DFS among the non-high-risk stage II (n = 60) and stage III (n = 139), and high-risk stage II (n = 179) and stage III (n = 173) subgroups.

B The impact of the cirScore on OS among the non-high-risk stage II (n = 60) and stage III (n = 139), and high-risk stage II (n = 179) and stage III (n = 173) subgroups.

C Time-dependent ROC analysis for predicting DFS and OS of stage II/III colon cancer patients in the three datasets using the clinical risk status + cirScore and clinical risk status only. AUC = area under the curve.

Data information: In (A, B), hazard ratios (HRs) were calculated with a univariate Cox regression analysis, and P-values were calculated with the log-rank test.

Figure EV5. Building nomograms and time-dependent ROC analysis.

A, B Calibration curve of 3-year DFS and 5-year DFS prediction in internal (n = 122) and external validation (n = 180) cohort. The 45-degree blue dotted line represents the reference line of an ideal nomogram. The error bars represent the 95% confidence intervals of the actual survival in the upper, middle, and lower tertiles.

C Nomogram to predict the OS of patients with stage II/III colon cancer.

D Calibration curves of the nomogram in prediction of the 3-year and 5-year OS in training (n = 249), and internal (n = 122) and external (n = 180) validation cohort. The 45-degree blue dotted line represents the reference line of an ideal nomogram. The error bars represent the 95% confidence intervals of the actual survival in the upper, middle, and lower tertiles. NI, perineural invasion. VI, lymphatic or vascular invasion.

© 2019 The Authors

© 2019 The Authors

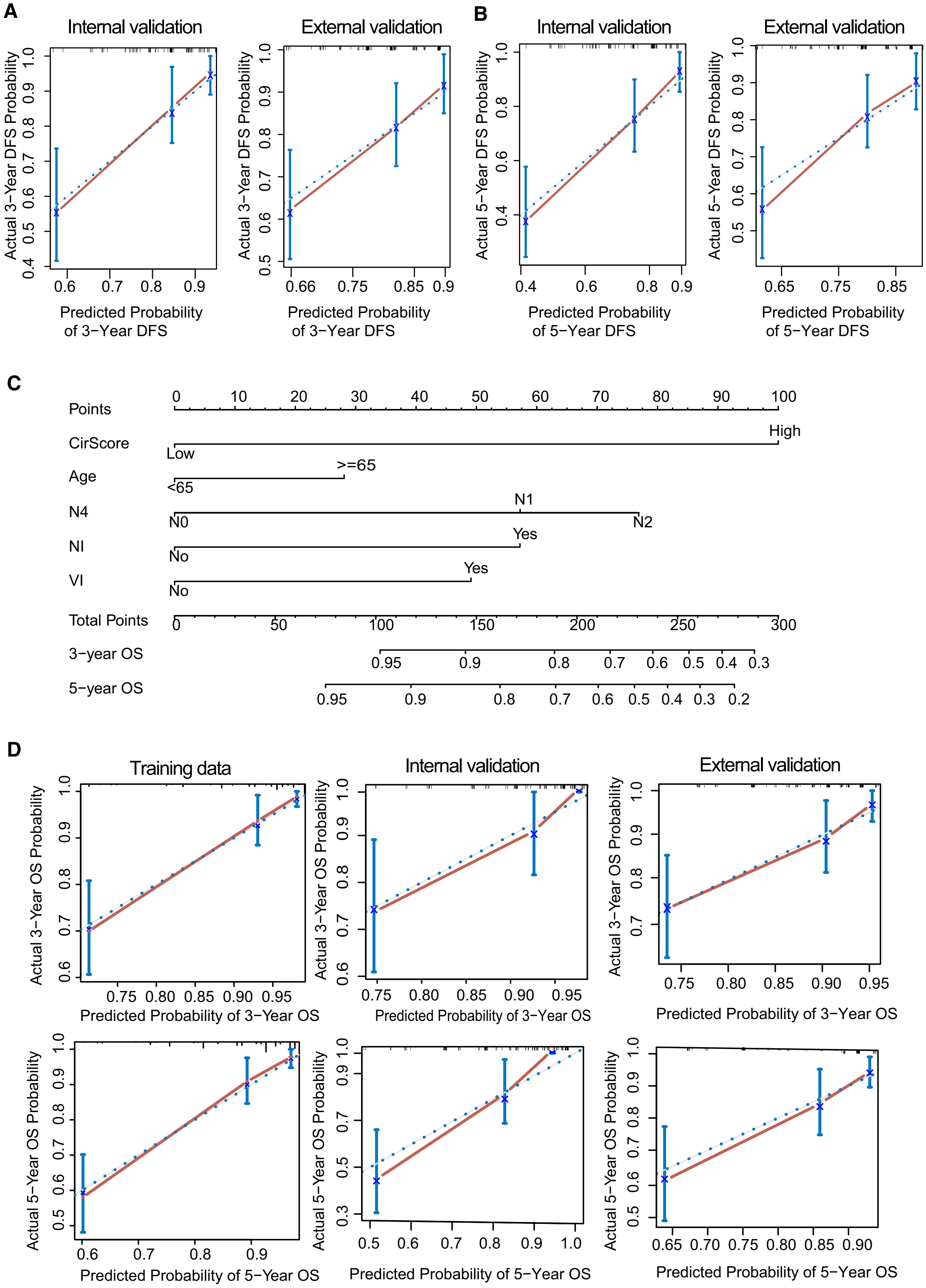

Figure EV5.

© 2019 The Authors
